# Cingulate Gyrus Volume as a Mediator of the Social Gradient in Cognitive Function

**DOI:** 10.31586/jcn.2025.1139

**Published:** 2025-01-24

**Authors:** Shervin Assari, Hossein Zare

**Affiliations:** 1 Department of Internal Medicine, Charles R. Drew University of Medicine and Science, Los Angeles, CA, United States; 2 Department of Family Medicine, Charles R. Drew University of Medicine and Science, Los Angeles, CA, United States; 3 Department of Urban Public Health, Charles R. Drew University of Medicine and Science, Los Angeles, CA, United States; 4 Marginalization-Related Diminished Returns (MDRs) Center, Los Angeles, CA, United States; 5 Department of Health Policy and Management, Johns Hopkins Bloomberg School of Public Health, Baltimore, MD, United States; 6 School of Business, University of Maryland Global Campus (UMGC), Adelphi, MD, United States

**Keywords:** Socioeconomic Status, Cognitive Function, Cingulate Gyrus, Mediation, ABCD Study, Sex Differences

## Abstract

**Background:**

Socioeconomic status (SES) is a well-established predictor of cognitive function in children, but the neurobiological pathways through which SES influences cognitive outcomes remain underexplored. This study examines the role of the cingulate gyrus (region of the brain that is involved in emotion regulation, decision-making, error detection, and cognitive control) in mediating the relationship between SES and cognitive performance, with a focus on whether these effects vary by sex.

**Objective:**

To investigate the role of the cingulate gyrus in mediating the association between social gradients (family SES) and cognitive function in children and assess potential sex differences in these pathways.

**Methods:**

Data were drawn from the Adolescent Brain Cognitive Development (ABCD) study. Cognitive function was assessed using a composite measure of executive function and general cognitive ability. Structural MRI data were used to measure the volume of the cingulate gyrus. Path analysis was conducted to examine the mediating role of the cingulate gyrus in the association between SES and cognitive function. Interaction terms were included to test for sex differences.

**Results:**

Higher SES was significantly associated with a larger cingulate gyrus volume and better cognitive function. The volume of the left cingulate gyrus partially mediated the relationship between family and neighborhood SES and cognitive function, explaining a portion of the social gradient in cognitive outcomes. No significant sex differences were found in these mediating effects.

**Conclusions:**

The cingulate gyrus partially mediates the link between SES and cognitive function in children. These findings suggest that social disparities in cognitive function may operate, in part, through neurobiological changes such as those in the cingulate gyrus, without significant variation by sex.

## 1. Introduction

Socioeconomic status (SES) is a critical determinant of children’s cognitive development [1–7]. Extensive research has shown that children from lower SES backgrounds are more likely to experience poor school performance, higher dropout rates, and lower cognitive function [8, 9]. In contrast, children from higher SES families generally display superior cognitive abilities and academic outcomes [8–13]. Although the link between SES and cognitive function is well-documented, the neurobiological mechanisms driving this relationship remain less understood [8, 14].

Emerging evidence suggests that children from disadvantaged backgrounds not only perform worse on cognitive tasks but may also exhibit structural differences in their brain development, particularly in regions of the cerebral cortex such as the frontal and parietal lobes [1, 8, 13]. These areas are essential for higher-order cognitive processes, including executive functioning and decision-making. One region of interest that may serve as a mediator of SES-related cognitive disparities is the cingulate gyrus, part of the brain that not only is involved in emotion regulation [limbic system] but also decision-making, error detection, and cognitive control [15].

The cingulate gyrus has been implicated in various cognitive functions, including attention, emotion processing, and error detection. In 1995, Devinsky, Morrell, and Vogt described that the anterior executive region of the cingulate is subdivided into ‘cognitive’ and ‘affective’ components. The cognitive division of the cingula includes caudal areas 24’ and 32’, the cingulate motor areas in the cingulate sulcus and nociceptive cortex. The nociceptive area is engaged in both response selection and cognitively demanding information processing. In contrast, the affect division includes areas 25, 33 and rostral area 24, and has extensive connections with the amygdala. It is involved in conditioned emotional learning, assessments of motivational content, and assigning emotional valence to internal and external stimuli [15]. In 2000, George Bush and colleagues published a review and discussed that while cingulate core is historically regarded as a part of the limbic system, responsible for affect and emotion regulation, neuroimaging research indicates that ACC was active in many studies of cognition. It is believed that cingulate cortex is involved in brain’s error detection and correction device [16].

Given its role in these processes, it is plausible that differences in the size or development of the cingulate gyrus could help explain why children from lower SES backgrounds tend to have poorer cognitive outcomes. Thus, investigating the cingulate gyrus as a potential mediator in the pathway from SES to cognitive function may shed light on the neurocognitive mechanisms underlying SES disparities in cognitive performance.

The present study aims to explore these indirect pathways by focusing on how SES influences cognitive function in a large, diverse sample of children. Using data from the Adolescent Brain Cognitive Development (ABCD) study [17–19], we apply structural equation modeling (SEM) [20–25] to test a mediational model that examines the indirect effects of SES on cognitive function through the cingulate gyrus volume. We hypothesize that lower SES will be associated with reduced cognitive function, partially mediated by smaller cingulate gyrus volume.

In addition to the SES-to-cognitive-function pathway, potential sex differences in this relationship merit exploration [26–30]. Previous research consistently shows that boys and girls follow different developmental trajectories in cognitive function across varying SES levels. Boys and girls may respond differently to environmental factors such as SES, with girls potentially being more vulnerable to environmental stressors. For example, girls may be more sensitive to social and emotional contexts, which can influence the development of brain regions like the cingulate gyrus. This suggests the possibility of sex-specific patterns in how SES shapes cognitive function, warranting a comparative analysis of boys and girls to determine whether these pathways differ by sex.

This study builds on existing literature that highlights SES as a key social determinant of health and cognitive development. By examining how SES, cingulate gyrus volume, and cognitive function interact—and whether these relationships differ between boys and girls—we aim to clarify the neurocognitive mechanisms by which SES disparities emerge during adolescence. Understanding these pathways in a large, diverse sample can provide valuable insights into the developmental processes that contribute to SES-related inequalities in cognitive outcomes and inform potential interventions aimed at mitigating these disparities.

## 2. Methods

### 2.1. Design and Sample

We conducted a secondary analysis using data from the Adolescent Brain Cognitive Development (ABCD) study [19, 31–39], a national longitudinal study of a racially and economically diverse cohort of pre-adolescent children. The ABCD study’s methodology has been thoroughly documented elsewhere. Advantages of the ABCD dataset include its longitudinal design, national scope, large and diverse samples in terms of race, SES, and geographic distribution. Participants were primarily recruited from schools.

### 2.2. Analytical Sample

The analytical sample consisted of participants across race, ethnic, and SES groups. Participants were 9–10-year-old at baseline. 11,878 children entered our analysis.

### 2.3. Ethics

The study was approved by the Institutional Review Board (IRB) of the University of California, San Diego (UCSD). Assent was obtained from all participating adolescents, and informed consent was obtained from their parents.

### 2.4. Study Variables

The study variables included race, demographic and socioeconomic factors, adversities, and substance use.

### 2.5. Predictors

#### 2.5.1. Confounders

##### Race:

Parents reported the race and ethnicity of their children. This was a categorical variable with White as the reference category.

##### Age:

Age at baseline was calculated in years. It was based on the number of years between birth and study date.

##### Sex:

This was a dichotomous variable with 1 for male and 0 for female.

##### Socioeconomic Status:

Socioeconomic status was defined a primary component analysis using the following variables (parental education, family income, financial stress, and family structure). This variable was then dichotomized (0 for high and 1 for low SES).

##### Parental Educational Attainment:

Participants were asked, “What is the highest grade or level of school you have completed or the highest degree you have received?” Responses were 0 = Never attended/Kindergarten only; 1 = 1st grade; 2 = 2nd grade; 3 = 3rd grade; 4 = 4th grade 4; 5 = 5th grade; 6 = 6th grade 6; 7 = 7th grade 7; 8 = 8th grade; 9 = 9th grade; 10 = 10th grade 10; 11 = 11th grade; 12 = 12th grade; 13 = high school graduate; 14 = GED or equivalent diploma; 15 = some college; 16 = associate degree: occupational; 17 = associate degree: academic program; 18 = bachelor’s degree (ex. BA; 19 = master’s degree (ex. MA; 20 = professional school degree (ex. MD; 21 = doctoral degree. This variable was an interval measure with a range between 1 and 21, with a higher score indicating higher educational attainment.

##### Family Income:

Family income was a continuous measure ranging from 1 to 10, with a higher score indicating higher income. The exact question was, “What is your total combined family income for the past 12 months? This should include income (before taxes and deductions) from all sources, wages, rent from properties, social security, disability and veteran’s benefits, unemployment benefits, workman”. Responses included 1 = Less than $5000; 2 = $5000; 3 = $12,000; 4 = $16,000; 5 = $25,000; 6 = $35,000; 7 = $50,000; 8 = $75,000; 9 = $100,000; 10 = $200,000.

##### Financial Stress:

Financial difficulties were assessed through financial difficulties experienced in the past 12 months. Items included inability to afford food, telephone service, rent/mortgage, eviction, utility shutoffs, and unmet medical or dental needs. Responses were binary (0 = no, 1 = yes), and a mean score was calculated, with higher scores indicating higher financial stress.

##### Family Structure:

Parents reported the number of parents in the household and their relationship. This was categorized as 0 for not married and 1 for married households.

### 2.6. Data Analysis

Data analysis was conducted using Stata. Univariate analysis involved reporting the mean and standard deviation (SD) of continuous measures. We used Pearson test to estimate bivariate correlations. Structural equation models (SEM) were used for multivariable analysis, with delinquency as the outcome. Predictor was heat wave. Race, SES, age, and gender were confounders. Collinearity between variables was checked and ruled out (all correlations were below .6). Beta, 95% confidence intervals (CI), and p-values were reported.

## 3. Results

The structural equation model (SEM) results are summarized in Table 1. The analysis revealed that the volume of the cingulate gyrus was significantly associated with cognitive function (B = 0.18, SE = 0.01, 95% CI [0.16, 0.19], p < 0.001), suggesting that greater cingulate gyrus volume is linked to better cognitive performance. Age also showed a strong positive association with cognitive function (B = 0.35, SE = 0.01, 95% CI [0.34, 0.36], p < 0.001), indicating that older children performed better on cognitive tasks.

Sex was a significant predictor of cognitive outcomes, with males showing lower cognitive performance compared to females (B = −0.05, SE = 0.01, 95% CI [−0.06, −0.03], p < 0.001). Both neighborhood education (B = 0.14, SE = 0.01, 95% CI [0.13, 0.16], p < 0.001) and parental education (B = 0.27, SE = 0.01, 95% CI [0.26, 0.29], p < 0.001) were positively associated with cognitive function, suggesting that children from higher-educated families and neighborhoods performed better cognitively. Living in a married household was also associated with higher cognitive function (B = 0.07, SE = 0.01, 95% CI [0.06, 0.09], p < 0.001).

Ethnic and racial differences were observed in cognitive performance. Black children had significantly lower cognitive function compared to White children (B = −0.14, SE = 0.01, 95% CI [−0.15, −0.12], p < 0.001), while Asian children showed a slight cognitive advantage (B = 0.02, SE = 0.01, 95% CI [0.01, 0.03], p = 0.005). Latino children did not differ significantly from White children in cognitive function (B = 0.00, SE = 0.01, 95% CI [−0.01, 0.02], p = 0.702).

Regarding predictors of cingulate gyrus volume, age was a strong positive predictor (B = 0.27, SE = 0.00, 95% CI [0.26, 0.28], p < 0.001), indicating that cingulate gyrus volume increased with age. Males had significantly larger cingulate gyrus volumes compared to females (B = 0.30, SE = 0.01, 95% CI [0.28, 0.31], p < 0.001). Both neighborhood education (B = 0.16, SE = 0.01, 95% CI [0.14, 0.18], p < 0.001) and parental education (B = 0.12, SE = 0.01, 95% CI [0.10, 0.14], p < 0.001) were positively associated with cingulate gyrus volume.

Racial and ethnic differences in cingulate gyrus volume were also evident. Black children had smaller cingulate gyrus volumes compared to White children (B = −0.06, SE = 0.01, 95% CI [−0.08, −0.04], p < 0.001), and Asian children had slightly smaller volumes (B = −0.04, SE = 0.01, 95% CI [−0.05, −0.02], p < 0.001). Latino children, on the other hand, had larger cingulate gyrus volumes compared to White children (B = 0.03, SE = 0.01, 95% CI [0.01, 0.05], p = 0.001).

Although living in a married household was marginally associated with larger cingulate gyrus volume (B = 0.02, SE = 0.01, 95% CI [0.00, 0.04], p = 0.051), this effect was not statistically significant at the 0.05 level. The “Other” race/ethnicity group showed a small but significant negative association with cingulate gyrus volume (B = −0.02, SE = 0.01, 95% CI [−0.03, 0.00], p = 0.042).

**Figure 1.**
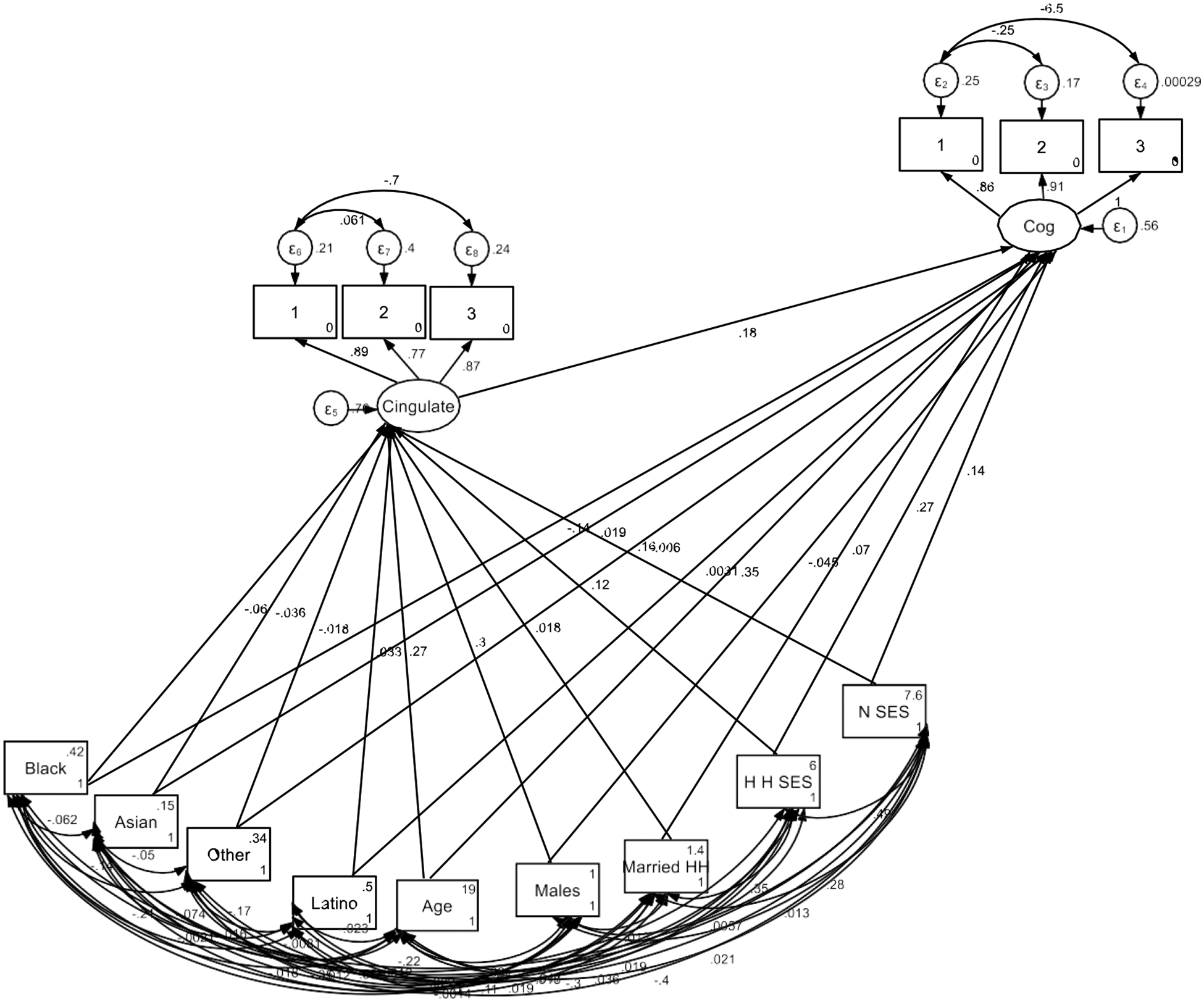
Summary of structural equation model (SEM)

Figure 1 summarizes the SEM findings. These findings suggest that the cingulate gyrus plays a significant role in the relationship between SES and cognitive function, with age, sex, and racial/ethnic background contributing to variations in brain structure and cognitive outcomes.

## 4. Discussion

The results of this study provide new insights into the neurobiological mechanisms that underlie the relationship between SES and cognitive function in children. Consistent with prior research, we found that SES was significantly associated with cognitive performance, supporting the well-documented influence of social gradients on cognitive development. More importantly, our findings suggest that the cingulate gyrus partially mediates this relationship, highlighting its potential role as a neuroanatomical pathway through which SES exerts its effects on cognition.

The cingulate gyrus is involved in a variety of cognitive processes, including attention, executive function, and emotional regulation. Its partial mediation of the SES-cognitive function relationship indicates that children from higher-SES backgrounds may benefit from more favorable neurodevelopmental trajectories, as reflected in the structure of the cingulate gyrus. These neurobiological advantages may help explain why higher SES is consistently linked to better cognitive outcomes.

Interestingly, our results showed no significant sex differences in these mediating effects. Although sex differences in cognitive development and brain structure have been reported in previous studies, the lack of variation in this context suggests that the influence of SES on cognitive outcomes through the cingulate gyrus is comparable for both boys and girls. This finding contrasts with research suggesting sex-specific neurodevelopmental patterns, particularly during adolescence, but aligns with studies showing that SES-related disparities in cognitive function tend to affect both sexes similarly.

The absence of major sex differences in the mediation by the cingulate gyrus has important implications. It suggests that interventions aimed at reducing SES-related cognitive disparities through neurobiological pathways, such as those targeting the development of brain regions involved in cognitive control, may be broadly effective across sexes. Future research could explore whether other brain regions or cognitive domains exhibit sex-specific mediating effects of SES, providing a more nuanced understanding of how social gradients influence neurodevelopment differently for boys and girls.

Several limitations should be considered when interpreting these findings. First, while the ABCD study provides a rich dataset for examining the neurobiological correlates of cognitive development, the cross-sectional nature of the data limits our ability to infer causal relationships between SES, brain structure, and cognitive function. Longitudinal studies are needed to clarify the directionality of these associations and to better understand how changes in SES over time might influence brain development and cognitive outcomes. Second, although we controlled for a number of confounding factors, there may be additional variables—such as parenting practices, environmental stressors, or genetic influences—that contribute to the observed relationships.

In conclusion, this study demonstrates that the cingulate gyrus plays a partial mediating role in the relationship between SES and cognitive function in children, providing evidence of a neurobiological pathway through which social disparities in cognition arise. The absence of significant sex differences in these effects suggests that interventions to reduce SES-related cognitive gaps may be similarly effective for boys and girls. Future research should continue to explore the neurobiological mechanisms underlying the SES-cognition link, with a focus on identifying potential targets for intervention and prevention.

## Figures and Tables

**Table 1.** Summary of SEM in the pooled sample

Predictor		Outcome	B	SE	95%	CI	p
Cingulate Area	→	Cognitive Function	0.18	0.01	0.16	0.19	< 0.001
Age	→	Cognitive Function	0.35	0.01	0.34	0.36	< 0.001
Male	→	Cognitive Function	−0.05	0.01	−0.06	−0.03	< 0.001
Neighborhood education	→	Cognitive Function	0.14	0.01	0.13	0.16	< 0.001
Parental Education	→	Cognitive Function	0.27	0.01	0.26	0.29	< 0.001
Married Household	→	Cognitive Function	0.07	0.01	0.06	0.09	< 0.001
Other	→	Cognitive Function	−0.01	0.01	−0.02	0.01	0.402
Latino	→	Cognitive Function	0.00	0.01	−0.01	0.02	0.702
Black	→	Cognitive Function	−0.14	0.01	−0.15	−0.12	< 0.001
Asian	→	Cognitive Function	0.02	0.01	0.01	0.03	0.005
Age	→	Cingulate Area	0.27	0.00	0.26	0.28	< 0.001
Male	→	Cingulate Area	0.30	0.01	0.28	0.31	< 0.001
Neighborhood education	→	Cingulate Area	0.16	0.01	0.14	0.18	< 0.001
Parental Education	→	Cingulate Area	0.12	0.01	0.10	0.14	< 0.001
Married Household	→	Cingulate Area	0.02	0.01	0.00	0.04	0.051
Other	→	Cingulate Area	−0.02	0.01	−0.03	0.00	0.042
Latino	→	Cingulate Area	0.03	0.01	0.01	0.05	0.001
Black	→	Cingulate Area	−0.06	0.01	−0.08	−0.04	< 0.001
Asian	→	Cingulate Area	−0.04	0.01	−0.05	−0.02	< 0.001

## Box 1. Variables for defining cognitive function and cingulate gyrus and sulcus area

**Table:**

**Cingulate Gyrus and Sulcus Area:**
mrisdp_309: left middle-anterior part of the cingulate gyrus and sulcus
mrisdp_310: left middle-posterior part of the cingulate gyrus and sulcus
mrisdp_308: left anterior part of the cingulate gyrus and sulcus
**Cognitive Function:**
Total Composite, Uncorrected
Picture Vocab, Uncorrected
Crystalized, Uncorrected
